# Factors Associated with Baseline CD4 Cell Counts and Advanced HIV Disease among Male and Female HIV-Positive Patients in Iran: A Retrospective Cohort Study

**DOI:** 10.1155/2022/8423347

**Published:** 2022-07-07

**Authors:** Sima Afrashteh, Mohammad Fararouei, Haleh Ghaem, Mohammad Aryaie

**Affiliations:** ^1^Student Research Committee, Shiraz University of Medical Sciences, Shiraz, Iran; ^2^HIV/AIDs Research Center, Shiraz University of Medical Sciences, Shiraz, Iran; ^3^Non-Communicable Diseases Research Center, Department of Epidemiology, School of Health, Shiraz University of Medical Sciences, Shiraz, Iran; ^4^Department of Epidemiology, School of Health, Shiraz University of Medical Sciences, Shiraz, Iran

## Abstract

Despite the recommendation for early diagnosis and rapid initiation of HIV treatment, more than half of patients are in an advanced stage of HIV disease in Iran. This study aimed to investigate the factors related to baseline CD4 cell count and advanced HIV disease (AHD) in Southern Iran. The study comprised all adults between 15 and 87 years of age who were newly diagnosed with HIV in Southern Iran. Linear and logistic regressions were used to identify baseline CD4 cell count predictors and AHD, respectively. A total of 820 (53.9%) HIV-infected individuals over 15 years of age were at the AHD stage. Based on the results of the multiple logistic regression, older age at diagnosis (OR_≥40/<30_ = 2.68, 95% CI = 1.38–5.19), gender (OR_female/male_ = 0.62, 95% CI = 0.44–0.85), HIV/TB coinfection (OR_yes/no_ = 1.98, 95% CI = 1.29–3.02), HIV/HBV coinfection (OR_yes/no_ = 1.58, 95% CI = 1.07–2.38), and hemoglobin (OR = 0.89, 95% CI = 0.85–0.92) were directly associated with AHD in HIV/AIDS patients. As suggested by a linear regression model, factors including gender (B _Female_ = 44.12, 95% CI:17.86, 70.38), older age (*B* _≥_ _40_ = −111.99, 95% CI:−174.70, −49.27), higher education level (*B* = 35.65, 95% CI:5.34, 65.97), WHO clinical stage (*B*_IV_ = −254.53, 95% CI−298.82, −210.24), and hemoglobin (*B* = 5.23, 95% CI:0.25, 10.20) were significantly associated with CD4 count.The prevalence of AHD in patients was high in Iran. Our results suggested that several demographic and clinical factors are significantly associated with the baseline CD4 cell count and AHD. Targeted HIV testing, implementation of screening programs for early detection, and access to care services to assure early ART are recommended to improve the clinical status and quality of life of the patients.

## 1. Introduction

HIV infection, with 37.7 million people already infected and approximately 1.5 million newly infected cases in 2020, is an imminent threat to human society and is one of the great health challenges of the present century worldwide [1]. Despite the global decline in the number of people living with HIV and the control of HIV epidemics in many developed countries, the rate of HIV infection is increasing in several developing countries such as Iran [2]. It is estimated that in Iran, by the end of 2020, 54,000 patients will be living with HIV, of which, only 29% of patients will receive antiretroviral treatment, a figure much less than the global target set by the Joint United Nations Programme on HIV/AIDS (UNAIDS) [3].

Among HIV patients, the CD4 cell count drops a few weeks after HIV infection and then increases again in the following months, yet it is less than the level before infection [4]. The CD4 cell count is considered one of the main indicators of the prognosis of HIV infection and patient survival [5]. A decrease in the number of these cells leads to an increase in the risk of mortality in AIDS patients [6]. Most importantly, the CD4 cell count is the best indicator of assessing the immune system status and the risk of opportunistic infections in HIV-infected patients [7]. The World Health Organization (WHO) has emphasized the use of CD4 counting in assessing early disease status, monitoring AIDS progression, making clinical decisions, and prioritizing care for advanced HIV patients [8]. The evidence shows an association between the CD4 cell count and coinfection with other infectious agents, life expectancy, response to antiretroviral therapy, AHD, and risk of death [7,9]. Old age, male gender, nutrition, clinical stage, geographical location, injection drug use (IDU), and unsafe sex are associated with lower baseline cd4 cell count [4, 8].

Despite the decline in the number of people living with HIV worldwide, mortality seems to be increasing due to the increasing incidence of advanced HIV disease [10]. Patients with advanced HIV disease are at risk of death due to reduced CD4 cell counts despite starting ART therapy [11]. Evidence suggests that even in access to treatment for all patients, a particular focus should be on AHD patients to reduce the high risk of HIV-related complications [12]. Prompt and early identification of patients with advanced HIV disease through CD4 testing is essential to reduce the burden of HIV in order to provide a specific treatment [13].

A drop in the number of CD4cells to less than 200 cells/mm^3^ and AHD expose HIV-infected patients to opportunistic infections and high mortality. In Iran, HIV-infected individuals are mainly diagnosed at an advanced stage of the disease with a poor prognosis [14]. As a result, examining the factors associated with CD4 cell count and AHD at the time of diagnosis is essential for guidance in making screening strategies and clinical decisions [15, 16]. Our better understanding of the HIV status at diagnosis has been shown to help in prolonging of life and potentially reducingthe risk of transmission of infection [17]. This study was conducted to identify the factors associated with the baseline CD4 cell count and AHD among HIV-positive patients in Southern Iran.

## 2. Methods

This is a cross-sectional presentation of the baseline measures of a retrospective cohort study, which was conducted from August 1997 to March 2021 among 1520 HIV-positive individuals in the Southern Iran. Behavioral diseases counseling centers (BDCC) collect the information of all diagnosed HIV patients in Southern Iran. In this center, after the first visit, patients are followed up for their disease progression and clinical status, and treatment once every six months. Also, after confirmation of the diagnosis, individuals are interviewed to complete a structured questionnaire including demographic characteristics, coinfection, high-risk behaviors, and drug use at the first visit. The trained staff of this center collect information via a face-to-face interview. Informed consent is routinely obtained from all patients at the first visit. The protocol of this study is approved by the Ethics Committee of Shiraz University of Medical Sciences (IR.SUMS.SCHEANUT.REC.1400.047).

### 2.1. Variables of Study

The predictor variables for this study were age group (<30, 30–39, ≥40), sex (male, female), educational level, marital status (single, married, widow/divorce), occupation (employed, unemployed), incarceration history (yes, no), addiction history (yes, no), possible route of transmission (injecting drugs (ID), sexual, mother to child/blood transfusion), HIV/TB coinfection (PPD diagnostic test, positive/negative), HIV/HCV coinfection (HCV Ab test, positive/negative), HIV/HBV coinfection (HbsAb and HbsAg tests, positive/negative), clinical stage of disease based on WHO definition (stage I, stage II, stage III, stage IV), and hemoglobin level (g/dl).

### 2.2. Definitions

In this study, advanced HIV disease (AHD) was defined as having a baseline CD4 cell count<200 or clinical stage 3 or 4 [18]. Inclusion criteria for initial CD4 cell count were patients having CD4 cell count (measured up to 6 months after diagnosis), and not receiving ART before determining the CD4 cell count [7, 19].

### 2.3. Statistical Analysis

Mean and standard deviation (SD) for continuous variables (age and CD4 count) are reported. For categorical variables, frequencies and percentages are reported. Univariate and multiple logistic regression models examined the crude and adjusted relationships between the study variables and the start of AHD. Also, univariate and multiple linear regression models were used to assess the factors associated with baseline CD4 cell counts. Variables with a *P*-value < 0.20 in univariate analysis were included in the final models. *P*-value < 0.05 was considered significant. STATA14.0 software (STATA, College Station, TX, USA) was used for all statistical analyses.

## 3. Result

Between August 1997 and March 2021, there were 1520 individuals newly diagnosed with HIV infection in Southern Iran. Of the newly diagnosed individuals, 820 (53.9%) were in an advanced HIV stage (of which, 102 (12.4%) were in stage 3 or 4 and 338 (41.2%) with the baseline CD4<200), and only 908 (59.7%) patients received a baseline CD4 cell count within six months after diagnosis. The mean age of participants in the study was 45.47 ± 9.49 years, and 71.4% were male. Most of the patients were unemployed (53.1%), and 47.7% were married. Also, 59.1% and 64.7% of the participants had a history of imprisonment and addiction, respectively. The most common route of transmission was IDU 690 (55.4%). According to the WHO clinical stage, 22.9% were in stage III, and 8.9% were in stage IV (Table 1).

### 3.1. Percentages of Advanced HIV Disease and Mean Baseline CD4 Cell Counts by the Transmission Group and Gender

**Figure 1:**
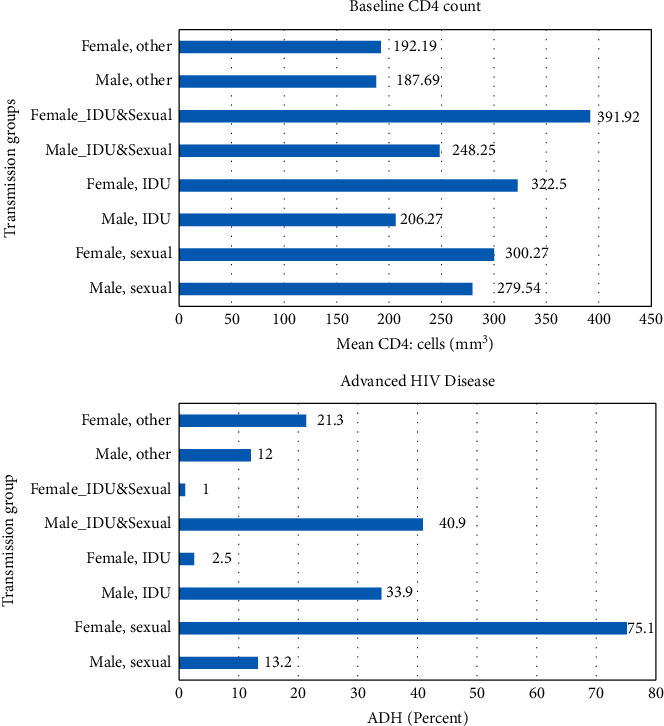
Percentages of advanced HIV disease and mean baseline CD4 from1997 to 2021 by transmission groups (sexual route, IDU, and others (mother to child, blood transition, and unknown)) and gender.

The percentages of advanced HIV disease and mean baseline CD4 by the main transmission group and gender are shown in Figure 1. Overall, compared with those infected via the sexual route and drug injection (IDU), those who are infected via IDU showed the lowest baseline CD4 count (205.3 ± 160.88). The highest percentage of AHD was reported among those infected via the sexual route, and among these patients, ADH was more common among female patients than in men (sexually infected females: 75.1 vs. sexually infected males: 13.2%). However, among IDU patients, men had a higher rate of advanced HIV disease than women (IDU-infected men: 33.9% vs. IDU infected women: 2.5%). Also, due to the much higher number of male patients who are mostly infected via IDU, AHD was more common in men than in women (76% vs. 24%) (Figure 1).

### 3.2. The Trend of Mean Baseline CD4 Cell Counts

**Figure 2:**
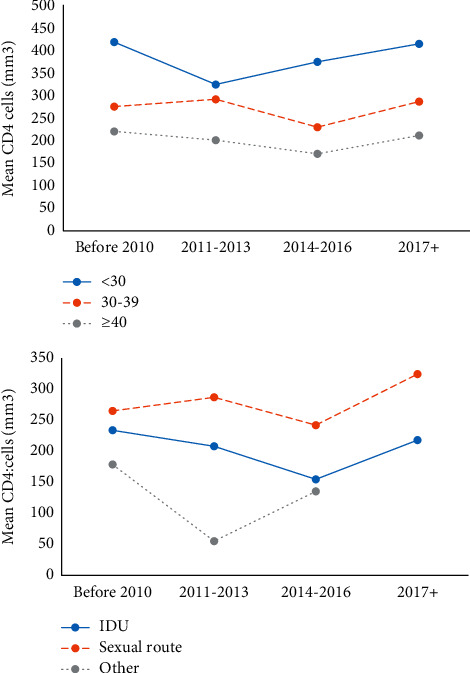
Changes of mean baseline CD4 cell counts from 1997 and 2021 stratified by age groups and transmission groups.

The trends of mean baseline CD4 cell counts during the study period (1997–2021) by the age groups and transmission groups are presented in Figure 2. Overall, the mean baseline CD4 cell counts for all age groups and the transmission groups increased from 1997 to 2021. However, the mean baseline CD4 cell counts for age groups 40 and older remained below 300 cells/ml. Subjects infected through IDU were consistently associated with lower mean baseline CD4 cell counts compared to those infected through sexual contact (Figure 2).

### 3.3. Factors Associated with the Baseline cd4 Cell Count

The crude and adjusted linear regression model regarding the association between the study factors and baseline CD4 cell counts are presented in Table 2. Based on the results of the univariate analysis, all the study variables, except marital status, were related to the number of CD4 cells . The results of multiple linear regression suggested that older individuals (age ≥ 40 years) had a significant negative effect on baseline CD4 cell counts (*B*_≥__40_ = −111.99, 95% CI:−174.70, −49.27, *P* < 0.001). The mean CD4 cell count was higher (B_Female_ = 44.12, 95% CI:17.86, 70.38, *P* = 0.001) among females. Higher education had a positive association with CD4 cell count (*B*_Academic_ = 35.65, 95% CI:5.34, 65.97, *P* = 0.021), *P* = 0.021). In addition, HIV/AIDS patients at II (B_II_ = −105.74,95% CI:−139.09, −72.40, *P* < 0.001), III (*B*_III_ = −215.59, 95% CI:−246.81, −184.36, *P* < 0.001), and IV (*B*_IV_ = −254.53, 95% CI−298.82, −210.24, *P* < 0.001) clinical stages experienced a lower CD4 cell count. In addition, a higher blood hemoglobin level was associated with a higher CD4 cell count (*B* = 5.23, 95% CI:0.25, 10.20, *P* = 0.039).

### 3.4. The Prognostic Factors with Advanced HIV Disease

The crude and adjusted logistic regression model for the association between the prognostic factors and advanced HIV disease is presented in Table 3. According to the univariate results, age, sex, education level, addiction history, IDU, blood hemoglobin level, TB, and HBV coinfection were significant prognostic factors for advanced HIV disease (*P* < 0.05). In addition, the multiple logistic regression results suggested that older patients who were 30–39 (OR_30-39/<30_ = 2.13, 95% CI = 1.08–4.22, *P* = 0.029) and ≥40 years old (OR_≥40/<30_ = 2.68, 95% CI = 1.38–5.19, *P* = 0.003) were at a higher risk of AHD than those who were younger. The risk of AHD for females was lower than in male patients (OR_female/male_ = 0.62, 95% CI = 0.44–0.85, *P* = 0.004). Patients infected through mother-to-child and blood transmission had 2.32 times higher odds of AHD than those sexually infected patients (OR_other/sexuallyroute_ = 2.32, 95% CI = 1.56–3.46, *P* < 0.001). TB coinfection (OR_yes/no_ = 1.98, 95% CI = 1.29–3.02, *P* = 0.001) and HBV coinfection (OR_yes/no_ = 1.58, 95% CI = 1.07–2.38, *P* = 0.022) were significantly associated with a higher odds of AHD. Finally, an increase in one g/dl of the blood hemoglobin level reduced the odds of AHD by 11% (OR = 0.89, 95%CI = 0.85–0.92, *P* < 0.001).

## 4. Discussion

To our best knowledge, this is the first study on the predictors of AHD in HIV-positive patients in Iran. This study aimed to identify the factors affecting the baseline CD4 cell count and AHD. We have shown that older age, sex, clinical stage, and hemoglobin level are the most important predictors of CD4 cell count and AHD in HIV patients. Coinfection with TB and HBV was also associated with AHD. This study estimated that the prevalence of AHD in patients from Southern Iran is 53.9%, suggesting the diagnosis and treatment of HIV as a serious problem in Iran. The prevalence is higher than the reported figure from China (40.1%) and lower than in Senegal (71%) [11, 20]. Diagnosis of HIV in Iran has clearly less quality than the goals defined by UNAIDS, which leads to the loss of opportunities for early diagnosis and treatment, and therefore diagnosing patients in the advanced stage with a poor prognosis [21].

The present study results showed that older age at diagnosis is an important predictor of CD4 cell count and AHD in HIV patients. In this study, older age had a negative effect on the baseline CD4 cell count. Means et al. showed that older people should start care in the early stages of the disease because the total immune reconstitution potential appears to decrease with age [5]. One of the reasons for a low CD4 cell count among the elderly is the late diagnosis of the disease due to lower education and literacy [7]. We also suggested that older people experience a higher risk of advanced HIV than younger people [11]. The elderlies need to be diagnosed early because the CD4 cells in these people are at a lower level, causing their immune systems to become more impaired and respond less effectively to ART. HIV causes more comorbidities and coinfections in older patients [4].

Another finding of our study was the role of gender in the baseline CD4 cell count and AHD. We showed that being a male has a negative effect on the number of CD4 cells and men are also more prone to develop AHD. Based on the available evidence, there seem to be conflicting results regarding gender differences in the progression of HIV. The results of several studies show that women have higher CD4 cell counts and a better immune function due to their higher perception of the risk of disease complications and seek more health care than men [9, 18, 22, 23]. However, Parsa et al. showed that CD4 + T-lymphocyte baseline values in women tend to decrease more rapidly, causing rapid progression toward advanced HIV infection in women compared to men. They believe that biological differences between the two genders may affect the natural history of HIV and the response to treatment [24]. These findings suggest a gender difference in the immune function, so continuous monitoring and follow-up of patients are recommended to better understand the underlying causes of the above differences between genders.

Consistent with the previous studies [25, 26], our study showed that a higher level of education is associated with a higher CD4 cell count. Higher education provides higher incomes, better nutrition, and a better understanding of health practices, which can increase CD4 levels in people living with HIV [26].

In line with the results of previous studies [7], our study, expectedly, showed that patients with advanced clinical stages (II, III, and IV) had significantly lower CD4 counts. In fact, the clinical stage of the disease can be used to assess the immunological status of HIV patients when CD4 counting facilities are not available [17].

We reported that HIV/TB co-infection increases the odds of AHD in the patients. In line with our results, Bruchfeld et al. showed that HIV and *Mycobacterium tuberculosis* infections strengthen each other, worsen immune function, and accelerate the progression from HIV to AIDS [6]. Co-occurrence of TB and HIV is a medical emergency, with the adverse outcomes of these infections including pancytopenia and low levels of CD4 and CD8, which contribute to the progression of AIDS [15]. Initiation of anti-TB treatment, as well as ART, management of drug cytotoxicity, and prevention/treatment of immune reconstitution inflammatory syndrome (IRIS), are essential for the clinical management of TB in HIV-infected patients [6].

Our study showed that HBV/HIV coinfection increases the odds of developing AHD in these patients. HBV/HIV coinfection is a major cause of cirrhosis, hepatocellular carcinoma, and advanced HIV (AIDS). Also, in these patients, due to the decrease in the number of CD4 cells, the specific immune response to HBV is disrupted [16]. Our findings are confirmed by another study [8].

The present study showed that hemoglobin as an important biomarker of disease progression is a predictor of CD4 cell count and AHD at baseline. Similarly, Qazi showed that low hemoglobin is strongly associated with decreased CD4 cell count in HIV patients [27]. The results of various studies showed that anemia in HIV patients, if stable, is associated with a significant reduction in the patient's survival. These studies have shown that a decrease in hemoglobin concentration is a reliable predictor of disease progression and higher mortality in HIV patients, so regular measures of this factor can help to determine which patients are at a higher risk for disease progression [28, 29]. Accordingly, low hemoglobin level is an adverse prognostic marker in HIV patients, and identifying and treating patients with anemia can improve the CD4 cell count, increase immune function, and delay disease progression.

### 4.1. Limitations

In our study, patients who were diagnosed from 1997 to 2021 and had their CD4 cell count measured within 6 months of diagnosis were included. According to WHO, a significant number of Iranian patients are not diagnosed/registered by the healthcare system and had no baseline measured. This means that the actual status of the patients from the southern part of Iran is possibly worse than what was reported in our study. Also, including registered cases from about 24 years ago may decrease the progressiveness of our conclusion.

## 5. Conclusion

The present study evaluated the baseline CD4 cell count, AHD, and their related factors. Our study showed that some demographic variables (age, gender, and education) and clinical variables (clinical stage, TB/HIV, and HBV/HIV coinfections) are associated with AHD and fewer CD4 cell counts in people with HIV from Southern Iran. The late diagnosis of the disease and the late initiation of ART cause a faster spread of HIV in the community. This is a major concern for the Iranian healthcare system. Therefore, the use of effective interventions such as targeted HIV testing, screening programs for early detection, among high-risk groups, and timely receipt of ART are recommended to reduce the burden of HIV in Iran.

## Figures and Tables

**Table 1:** Analysis of the demographic and clinical of HIV-positive patients according to gender.

Characteristics	Categories	Total number of patients (*N* = 1520)	Male (1086)	Female (434)
		No. (%)	No. (%)	No. (%)
Age at diagnosis (years)	<30	48(3.2)	26(2.4)	22(5.1)
	30–39	344(22.6)	222(20.4)	122(28.1)
	≥40	1128(74.2)	838(77.2)	290(66.8)

Education (at diagnosis)	Illiterate and primary	530(34.9)	369(34.0)	161(37.1)
	Secondary school	569(37.4)	444(40.9)	125(28.8)
	High school and diploma	335(22.0)	229(21.1)	106(24.4)
	Academic	86(5.7)	44(4.1)	42(9.7)

Marital status	Married	725(47.7)	475(43.7)	250(57.6)
	Single	387(25.5)	377(34.7)	10(2.3)
	Widowed/divorced	408(26.8)	234(21.5)	174(40.1)

Occupation	Employed	713(46.9)	657(60.5)	56(12.9)
	Unemployed	807(53.1)	429(39.5)	378(53.1)

Incarceration history (at diagnosis)	Yes	899(59.1)	871(80.2)	28(6.5)
	No	621(40.9)	215(19.8)	406(93.5)

Addiction history	Yes	983(64.7)	932(85.8)	51(11.8)
	No	537(35.3)	154(14.2)	383(88.2)

TB coinfection	Yes	127(8.4)	117(10.8)	10(2.3)
	No	1393(91.6)	969(89.2)	424(97.7)

HCV coinfection	Yes	761(50.1)	728(67.)	33(7.6)
	No	759(49.9)	358(33.0)	401(92.4)

HBV coinfection	Yes	142(9.3)	126(11.6)	16(3.7)
	No	1378(90.7)	960(88.4)	418(96.3)

Advanced HIV disease	Yes	820(53.9)	623(57.4)	197(45.4)
	No	700(46.1)	463(42.6)	237(54.6)

WHO clinical stage	I	686(45.1)	466(42.9)	220(50.7)
	II	351(23.1)	262(24.1)	89(20.5)
	III	348(22.9)	257(23.7)	91(21.0)
	IV	135(8.9)	101(9.3)	34(7.8)

Route of transmission	Sexual route	511(33.6)	153(14.1)	358(82.5)
	Injection drug use	842(55.4)	823(75.8)	19(4.4)
	Others^*∗*^	167(11.0)	110(10.1)	57(13.1)

Baseline CD4 count		251.34 ± 199.80	236.90 ± 187.85	287.41 ± 223.11

Hemoglobin		13.48 ± 2.79	13.58 ± 3.06	13.25 ± 1.93

^*∗*^Other modes of transmission included mother to child, blood transition, and unknown.

**Table 2:** Risk factors associated with baseline CD4 (linear regression model), Iran, 1997–2021.

Variables	Categories	Unadjusted coefficient (95% CI)	*P*-value	Adjusted coefficient (95% CI)	*P*-value
Age at diagnosis (years)	<30	1	—	1	—
	30–39	−127.65(−194.63, −60.46)	<0.001	−77.86(−142.31, −13.41)	**0.018**
	≥40	−200.53(−264.44, −136.62)	<0.001	−111.99(−174.70, −49.27)	**<0.001**

Sex	Male	1	—	1	—
	Female	68.57(39.92, 97.23)	<0.001	44.12(17.86, 70.38)	**0.001**

Education (at diagnosis)	Illiterate and primary	1	—	1	—
	Secondary school	25.69(−7.89, 59.27)	0.134	35.65(5.34, 65.97)	**0.021**
	High school and diploma	15.73(−21.79, 53.26)	0.411	−1.98(−35.72, 31.76)	0.908
	Academic	79.51(23.56,135.46)	0.005	39.60(−11.75, 90.96)	0.131

Marital status	Married	1	—	1	—
	Single	−9.22(−44.79, 26.34)	0.611	—	—
	Widowed/divorced	−22.02(−55.38, 11.33)	0.195	—	—

Occupation	Employed	1	—	1	—
	Unemployed	19.62(−8.57, 47.83)	0.172	—	—

Incarceration history	No	1	—	1	—
	Yes	−36.60(−64.51, −8.69)	0.010	—	—

History of addiction	No	1	—	1	—
	Yes	−53.89(−81.74, −26.03)	<0.001	—	—

Route of transmission	Sexual route	1	—	1	—
	Injection drug use	−83.47(−113.18, −53.76)	<0.001	—	—
	Others	−100.95(−142.10, −59.81)	<0.001	—	—

TB coinfection	No	1	—	1	—
	Yes	−106.78(−167.66, −45.90)	0.001	—	—

HCV coinfection	No	1	—	1	—
	Yes	−36.08(−65.00, −7.15)	0.015	—	—

HBV coinfection	No	1	—	1	—
	Yes	−97.14(−144.97, −49.32)	<0.001	—	—

WHO clinical stage	I	1	—	1	—
	II	−118.54(−150.98, −86.11)	<0.001	−105.74(−139.09, −72.40)	**<0.001**
	III	−235.88(−265.74, −206.02)	<0.001	−215.59(−246.81, −184.36)	**<0.001**
	IV	−268.94(−309.12, −228.75)	<0.001	−254.53(−298.82, −210.24)	**<0.001**

Hemoglobin		12.31(6.68, 17.93)	<0.001	5.23(0.25, 10.20)	**0.039**

**Table 3:** Adjusted association of study variables with advanced HIV disease, Iran, 1997–2021.

Variables	Categories	Unadjusted odds ratio (95% CI)	*P*-value	Adjusted odds ratio (95% CI)	*P*-value
Age at diagnosis (years)	<30	1	—	1	—
	30–39	2.26(1.17–4.37)	0.015	2.13(1.08–4.22)	**0.029**
	≥40	3.18(1.69–6.00)	<0.001	2.68(1.38–5.19)	**0.003**

Sex	Male	1	—	1	—
	Female	0.61(0.49–0.77)	<0.001	0.62(0.44–0.85)	**0.004**

Education (at diagnosis)	Illiterate and primary	1	—	1	—
	Secondary school	0.84(0.66–1.06)	0.159	—	—
	High school and diploma	0.94(0.71–1.24)	0.690	—	—
	Academic	0.57(0.36–0.91)	0.020	—	—

Marital status	Married	1	—	1	—
	Single	1.14(0.89–1.47)	0.272	-	—
	Widowed/divorced	1.24(0.97–1.59)	0.074	-	—

Occupation	Employed	1	—	1	—
	Unemployed	1.00(0.82–1.23)	0.947	—	—

Incarceration history	No	1	—	1	—
	Yes	1.25(1.02–1.54)	0.028	—	—

History of addiction	No	1	—	1	—
	Yes	1.37(1.11–1.70)	0.003	—	—

Route of transmission	Sexual route	1	—	1	—
	Injection drug use	1.56(1.25–1.95)	<0.001	0.95(0.68–1.31)	0.760
	Others	2.85(1.96–4.15)	<0.001	2.32(1.56–3.46)	**<0.001**

TB coinfection	No	1	—	1	—
	Yes	2.50(1.66–3.76)	<0.001	1.98(1.29–3.02)	**0.001**

HCV coinfection	No	1	—	1	—
	Yes	1.02(0.83–1.25)	0.800	—	—

HBV coinfection	No	1	—	1	—
	Yes	2.04(1.40–2.94)	<0.001	1.58(1.07–2.38)	**0.022**

Hemoglobin		0.88(0.85–0.91)	<0.001	0.89(0.85–0.92)	**<0.001**

## Data Availability

The data of this study is not publicly available due to its being the intellectual property of Shiraz University of Medical Sciences but is available from the corresponding author on a reasonable request.
